# A comparison of the risk of congestive heart failure-related hospitalizations in patients receiving hemodialysis and peritoneal dialysis - A retrospective propensity score-matched study

**DOI:** 10.1371/journal.pone.0223336

**Published:** 2019-10-01

**Authors:** Chien-Yao Sun, Junne-Ming Sung, Jung-Der Wang, Chung-Yi Li, Yi-Ting Kuo, Chia-Chun Lee, Jia-Ling Wu, Yu-Tzu Chang

**Affiliations:** 1 Department of Internal Medicine, National Cheng Kung University Hospital, College of Medicine, National Cheng Kung University, Tainan, Taiwan; 2 Department of Public Health, National Cheng Kung University, College of Medicine, Tainan, Taiwan; 3 Department of Environmental and Occupational Health, National Cheng Kung University Hospital, Tainan, Taiwan; University of Mississippi Medical Center, UNITED STATES

## Abstract

**Introduction:**

Congestive heart failure (CHF) is associated with high mortality and a heavy financial and healthcare burden in the dialysis population. Determining which dialysis modality is associated with a higher risk of developing CHF might facilitate clinical decision making and surveillance programs in the dialysis population.

**Methods:**

Using the Taiwan National Health Insurance Database, we recruited all incident dialysis patients during the period from January 1, 1998 to December 31, 2010. The propensity score matching method was applied to establish the matched hemodialysis (HD) and peritoneal dialysis (PD) cohort. Incidence rates and cumulative incidence rates of CHF-related hospitalization were first compared for the HD and PD patients. Multivariable subdistribution hazards models were then constructed to control for potential confounders.

**Results:**

Among a total of 65,899 enrolled dialysis patients, 4,754 matched pairs of HD and PD patients were identified. The incidence rates of CHF in the matched HD and PD patients were 25.98 and 19.71 per 1000 patient-years, respectively (P = 0.001). The cumulative incidence rate of CHF was also higher in the matched HD patients (0.16, 95% confidence interval (CI)(0.12–0.21)] than in the corresponding PD patients (0.09, 95% CI [0.08–0.11])(P<0.0001). HD was consistently associated with an increased subdistribution hazard ratio (HR) of CHF compared with PD in the matched cohort (HR: 1.45, 95% CI [1.23–1.7]). Similar phenomenons were observed in either the subgroup analysis stratified by selected confounders or in the HD and PD group without matching.

**Conclusions:**

HD is associated with a higher risk of developing CHF-related hospitalization than PD. The surveillance program for CHF should differ in patients receiving different dialysis modalities.

## Introduction

Despite tremendous advances in the management of congestive heart failure (CHF) in the past two decades, CHF is still prevalent in end-stage renal disease (ESRD) patients on maintenance dialysis therapy and is one of the leading causes of mortality in the dialysis population [1, 2]. It is estimated that the incidence of CHF can be 1.9-304-fold higher in the ESRD population as compared to the non-ESRD population across various age stratifications [3]. The development of CHF in the dialysis population results in further increases in health-care and financial burdens [4–7] as well as a higher frequency of hospitalization [1, 8, 9]. It is speculated that the choice of dialysis modality might lead to different degrees of susceptibility to the development of CHF mainly because hemodialysis (HD) and peritoneal dialysis (PD) are quite different with respect to uremic toxin clearance [10], hemodynamic stability during the dialysis process [11], and preservation of residual renal function [12], all of which may differentially affect the incidence of CHF. In Taiwan, the PD utilization rate increased gradually from 6.8% in 2000 to 8.8–9.5% during 2011–2015. Since patients receiving PD therapy have been proven to have a similar life expectancy but lower healthcare expenditures than those receiving HD therapy [13], Taiwan’s government launched a series of regulations to promote the utilization of PD, including the guarantee of a fixed amount of reimbursement payment to PD therapy and the setting of the utilization rate of PD being over 15% in medical centers as a criterion in the hospital accreditation system evaluation. Therefore, an increase in the prevalence of PD therapy also helps enhance patient literacy concerning the differential risks of CHF among the various dialysis modalities. Most previous studies have exclusively investigated the interrelationship between dialysis modality and *de novo* cardiovascular events or cardiovascular mortality [14–16]. Only one study has specifically investigated the role of dialysis modality in the development of incident CHF, but it had a limitation of potential dialysis modality selection bias [17]. In this study, we conducted an analysis by constructing a propensity score-matched cohort, which has the potential to minimize dialysis modality selection bias and to also facilitate the clarification of whether the risk of CHF differs between HD or PD therapy.

## Materials and methods

### Data source

This nationwide cohort study was constructed from the National Health Insurance Research Database (NHIRD), which is a large-scale computerized database including all kinds of reimbursement data related to National Health Insurance (NHI) in Taiwan. The NHI program was launched March 1, 1995 and covered more than 99% of the Taiwanese population by the end of 2014 [18]. It reimburses for nearly every kind of medical service, including payments for inpatient and outpatient services, medications, and intervention procedures. The NHI system sets forth a list of catastrophic illnesses, including ESRD patients on maintenance dialysis therapy, for which patient copayments can be waived to reduce their financial burden. The certification of a catastrophic illness for each patient must be evaluated by experts to avoid abuse. Furthermore, the waiving of copayments can also improve the adherence of these patients to the NHI system. This study was conducted after the approval by the Institutional Review Board (IRB) of the National Cheng Kung University Hospital (A-ER-101-089). The requirement of written informed consent was waived by the IRB because patient identification information had been encrypted before releasing the database to the researchers.

### Study design and identification of study population

**Fig 1:**
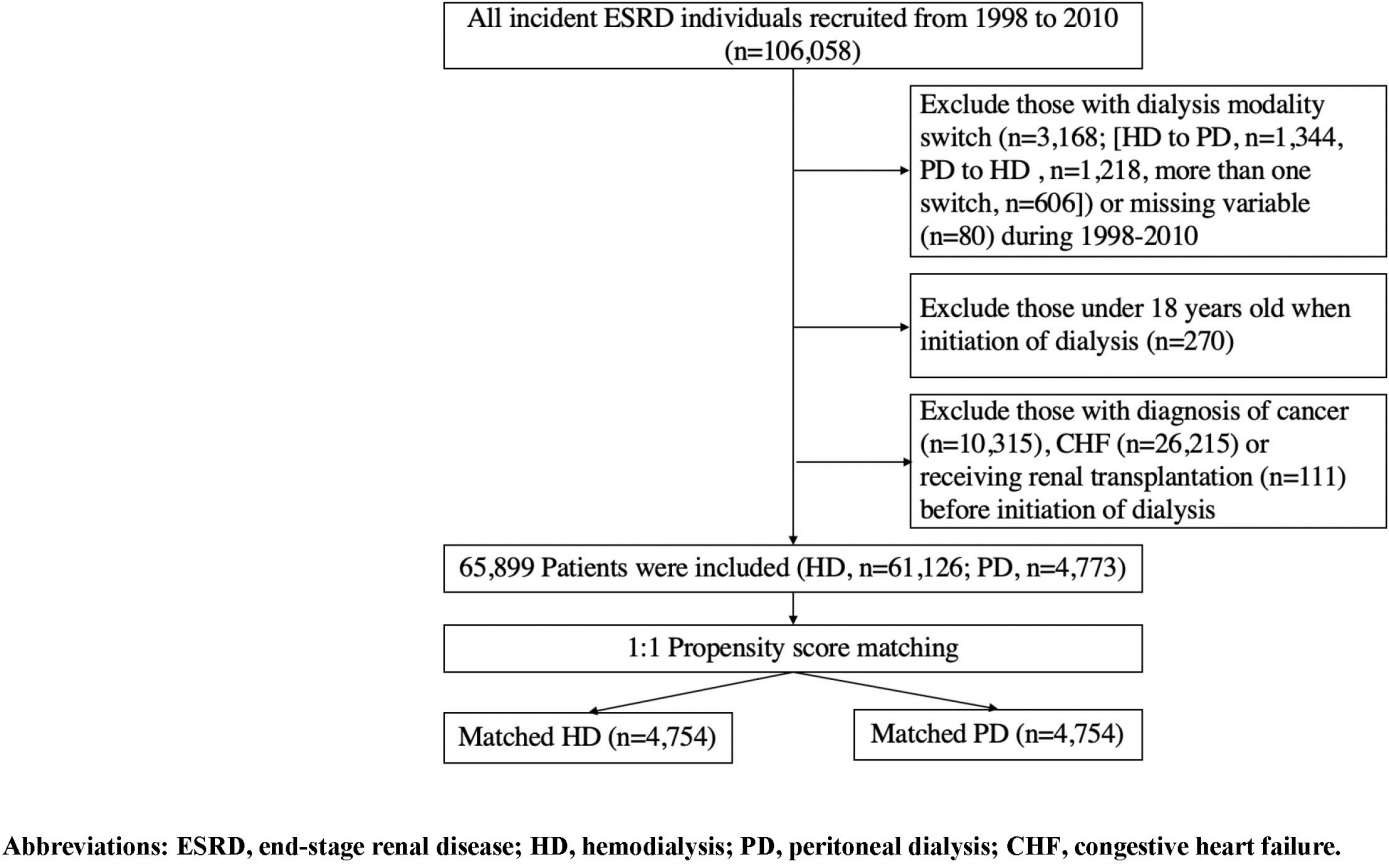
Flow chart of the establishment of matched-pairs of dialysis patients receiving hemodialysis or peritoneal dialysis.

From the NHIRD, we first identified all incident ESRD patients who received dialysis therapy in three or more consecutive months with certifications of catastrophic illness related to dialysis therapy (HD or PD) from January 1, 1998 to December 31, 2010. Patients aged less than 18 years at the initiation of dialysis, with missing variables, malignancies, dialysis-modality switching for three or more consecutive months in the period from 1998–2010, experiencing CHF requiring hospital care, or receiving transplantation before initiation of dialysis therapy were excluded (Fig 1). Patients with missing variables (n = 80), which represented only 0.075% of the whole dialysis population, were excluded from the final analysis because the exclusion of such a small number of patients would not comprise the representativeness of the dialysis cohort. The date of the enrollment was the date on which dialysis was initiated. The identification of HD and PD therapies was determined according to the procedure codes in the NHI program.

### Identification of incidence of congestive heart failure, comorbidities, and concomitant use of medication at baseline

We identified the incidence of CHF (International Classification of Diseases, Ninth Revision, Clinical Modification (ICD-9-CM) codes 398.91, 425, 428, 402.X1, 404.X1, and 404.X3) from the inpatient claim data, which indicated that only patients with CHF symptoms requiring hospitalization for further management would be treated as reaching the endpoint of our study. The date of endpoint occurrence was set as the first day of hospitalization for CHF. The following comorbidities were considered as potential confounders in the study: diabetes, hypertension, valvular heart disease, coronary artery disease, acute myocardial infarction (AMI), hyperlipidemia, anemia, chronic obstructive lung disease, and alcoholism (S1 Table) [19]. Patients were considered as having a specific comorbidity mentioned above if this specific condition was present once in the discharge codes or at least twice in ambulatory care visits 30 days apart in one year before the initiation of dialysis therapy. Patients who withdrew from the NHI program due to mortality or did not have medical claims for dialysis therapy on more than 60 consecutive days and without having any subsequent medical service reimbursed by the NHI were considered dead. Therefore, the day of NHI withdrawal or 30 days after dialysis therapy was discontinued was defined as the date of mortality. Furthermore, several types of medications have been found to be associated with a lower risk of CHF, including angiotensin-converting-enzyme inhibitors (ACEIs), angiotensin receptor blockers (ARBs), aldosterone antagonists, β-blocker, diuretic, digoxin, and the combined use of nitrate and hydralazine [19]. Therefore, patients who had received any of the above medications within one year before the initiation of dialysis were viewed as users of these medications. Patients were treated as censored if they withdrew from the NHI program not due to mortality, on the date of receiving kidney transplantation during the follow-up period, or at the end of the study period, December 31, 2010.

### Matching of patients with HD and PD based on the propensity score model

In order to balance the distribution of potential confounders and to minimize the potential indication bias between HD and PD patients, a 1:1 matching of incident HD and PD patients from the national dialysis cohort was performed according to their corresponding propensity scores. The propensity score for a PD prescription was estimated using a logistic regression model [20, 21], which included age, sex, index year of initiation of dialysis therapy, all comorbidities, and concomitant use of the medications listed in Table 1 as the independent variables. These variables were chosen for matching because of their association with CHF. This is based on the theory that the inclusion of variables that can affect outcome (CHF in our study) rather than treatment (HD or PD in our study) in a propensity score model is beneficial because they minimize the variance in the estimated treatment effect [22]. We constructed matched pairs according to the nearest neighbor matching method algorithm, which randomly selected each PD patient and matched the nearest score of a corresponding HD patient with a caliper width ranging between -0.1 and +0.1.

**Table 1:** Comparison of demographic and clinical characteristics of dialysis patients before and after matching by propensity score.

	Before matching			After matching		
	All HD patients	All PD patients	*P* value	Matched HD patients	Matched PD patients	di
**Number of patients**	61126	4773		4754	4754	
**Age, No. (%)**						
Mean (SD)	60.45 (14.16)	52.60 (14.86)	<0.0001	52.38 (14.83)	52.70 (14.78)	2.16
18–34 years	2669 (4.37)	600 (12.57)		621 (13.06)	581 (12.22)	
35–49 years	11253 (18.41)	1350 (28.28)		1384 (29.11)	1350 (28.4)	
50–64 years	21145 (34.59)	1775 (37.19)		1680 (35.34)	1775 (37.34)	
65–79 years	21276 (34.81)	869 (18.21)		919 (19.33)	869 (18.28)	
≥ 80 years	4783 (7.82)	179 (3.75)		150 (3.16)	179 (3.77)	
**Sex (No. of males, %)**	31461 (51.47)	2242 (46.97)	<0.0001	2288 (48.13)	2239 (47.1)	2.06
**Index year**			<0.0001			2.61
1998–2001	16919 (27.68)	133 (2.79)		149 (3.13)	133 (2.8)	
2002–2005	18488 (30.25)	436 (9.13)		458 (9.63)	436 (9.17)	
2006–2010	25719 (42.08)	4204 (88.08)		4147 (87.23)	4185 (88.03)	
**Duration of follow-up (years)**	4.01 (3.24)	2.55 (1.88)	<0.0001	2.89 (2.13)	2.55 (1.88)	16.92
**Baseline Comorbidities (%)**						
Diabetes mellitus	31161 (50.98)	1744 (36.54)	<0.0001	1685 (35.44)	1744 (36.68)	2.58
Hypertension	50655 (82.87)	3908 (81.88)	0.0801	3907 (82.18)	3893 (81.89)	0.77
Coronary artery disease	14507 (23.73)	918 (19.23)	<0.0001	905 (19.04)	918 (19.31)	0.69
Acute myocardial infarction	1990 (3.26)	124 (2.6)	0.0130	111 (2.33)	124 (2.61)	1.76
Anemia	53917 (88.21)	4109 (86.09)	<0.0001	4175 (87.82)	4096 (86.16)	4.94
Hyperlipidemia	25894 (42.36)	2256 (47.27)	<0.0001	2265 (47.64)	2247 (47.27)	0.76
Alcoholism	1247 (2.04)	67 (1.4)	0.0025	57 (1.2)	67 (1.41)	1.85
Chronic obstructive lung disease	10999 (17.99)	614 (12.86)	<0.0001	586 (12.33)	614 (12.92)	1.77
Valvular heart disease	3246 (5.31)	228 (4.78)	0.1122	185 (3.89)	227 (4.77)	4.34
**Baseline Medication (%)**						
ACEI	23611 (38.63)	1340 (28.07)	<0.0001	1273 (26.78)	1338 (28.14)	3.06
ARB	21973 (35.95)	2501 (52.4)	<0.0001	2399 (50.46)	2484 (52.25)	3.58
Aldosterone antagonists	4952 (8.1)	242 (5.07)	<0.0001	189 (3.98)	242 (5.09)	5.36
Beta-blocker	34411 (56.3)	2926 (61.3)	<0.0001	2917 (61.36)	2909 (61.19)	0.35
Diuretics	39334 (64.35)	2763 (57.89)	<0.0001	2712 (57.05)	2753 (57.91)	1.74
Digitalis glycosides	58 (0.09)	0 (0)	0.0210	0 (0)	0 (0)	-
Nitrate + hydralazine	1714 (2.8)	72 (1.51)	<0.0001	55 (1.16)	72 (1.51)	3.11

Abbreviations: di, standardized differences; ACEI, angiotensin-converting-enzyme inhibitor; ARB, angiotensin II receptor blockers.

### Statistical analysis

The continuous variables were summarized as means and standard deviations, and the categorical variables were listed as the number of cases and percentages. The comparison of continuous between-group variables was done through the use of a Student’s t-test, and the categorical variables were assessed with either a Chi-square test or a Fisher’s exact test. The estimation of incidence rate was calculated under the Poisson assumption. After matching based on the propensity score, the standardized differences were used to assess the differences in the baseline covariates between the study groups. Because of the high mortality rate of dialysis patients and a long follow-up period adopted in our study, the estimation of cumulative incidence rates was based on the cumulative incidence competing risk analysis, and the difference between the HD and PD groups was compared by using the modified Gray’s test [23, 24]. Furthermore, hazard ratios were estimated from Cox proportional subdistribution hazard regression models. The assumption of proportional sub-distribution hazards in Cox regression models was checked by *log(-log(survival function))* versus log of survival time graph, according to each of the covariates. A forest plot was used to present the hazard ratios of the subgroup population stratified using the selected potential confounders. Tests for interaction between dialysis modalities and selected covariates were assessed in the Cox regression models. All statistical analyses were performed with SAS version 9.4 (SAS Institute, Cary, NC.). Two-sided *p* values < 0.05 were defined as statistically significant in this study.

## Results

### Baseline characteristics of the study cohort before and after matching

We first identified 65,899 incident dialysis patients from 1998 to 2010, of which 61,126 patients received HD, and 4,773 patients received PD (Fig 1). Among the unmatched cohort, the HD patients were older, predominantly male, had more comorbidities, with the exception of hyperlipidemia, and had higher proportions of receiving ACEI, aldosterone antagonists, diuretics, digitalis, and nitrate + hydralazine (Table 1). The preliminary analysis of the concordance statistics of the logistic regression used to create the propensity scores was 0.83. We then performed 1:1 matching of the incident HD and PD patients based on the propensity scores, and 4,754 matched pairs of dialysis patients were successfully identified. The distribution of baseline characteristics and the use of selected medications were similar in the matched pairs of the HD and PD patients (Table 1).

### Comparison of overall, age- and sex-specific incidence rates and cumulative incidence rates of CHF between the HD and PD patients with and without matching

**Fig 2:**
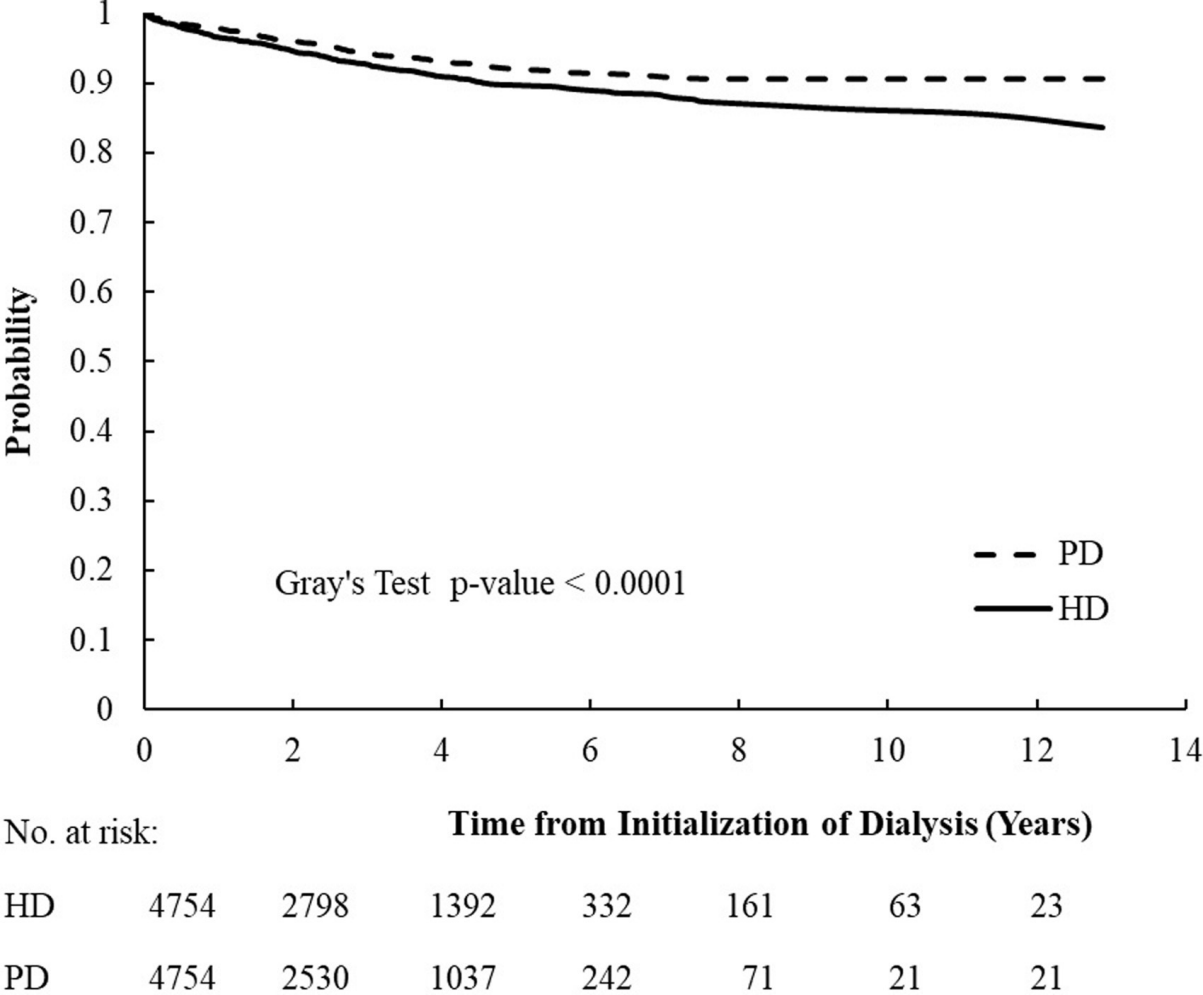
Comparison of cumulative congestive heart failure free survival rates between matched pairs of hemodialysis (HD) and peritoneal dialysis (PD) patients after accounting for competing risk of mortality.

The CHF incidence rates among the matched and unmatched cohorts stratified by age and sex as well the cumulative incidence competing risk analysis are presented in Table 2. Among the matched cohort, the mean follow-up duration for HD and PD patients were 2.89 and 2.55 years, respectively. The crude overall incidence rate of CHF per 1,000 patient-years was 25.98 (95% CI: 23.35–28.82) in the HD group, which was significantly higher than that of the PD group (19.71 [95% CI: 17.29–22.38]). When stratified by age and sex, the incidence increased incrementally with aging in both genders. A comparison of the incidence rate between the matched HD and PD groups in each age spectrum indicated that HD was almost always associated with a higher risk of CHF than PD. The cumulative incidence rates for the HD patients was also significantly higher in the HD patients than it was in the PD patients (0.16 [95% CI:0.12–0.21] vs. 0.09 [95% CI: 0.08–0.11], respectively, *p* value < 0.0001) (Table 2 and Fig 2).

**Table 2:** Comparison of incidence rates (per 1,000 patient-years) and subdistribution hazard ratios of congestive heart failure between patients with hemodialysis (HD) and peritoneal dialysis (PD) before and after matching for propensity score.

Characteristics	Before matching					After matching				
	All HD patients		All PD patients			Matched HD patients		Matched PD patients		
	No. of events	Incidence rates	No. of events	Incidence rates	aSHR^a^ (95% CI) (Ref. = PD)	No. of events	Incidence rates	No. of events	Incidence rates	aSHR^a^ (95% CI) (Ref. = PD)
**Overall**	8,342	34.05 (33.32–34.79)^d^	239	19.63 (17.22–22.28)^d^	1.54 (1.35–1.75)^b^	357	25.98 (23.35–28.82)^d^	239	19.71 (17.29–22.38)^d^	1.45 (1.23–1.70)^b^
**Male**					1.53 (1.27–1.85)^b^					1.44 (1.14–1.83)^c^
**Age (years)**										
18–34 years	127	13.25 (11.05–15.77)	6	7.69 (2.82–16.74)		13	12.03 (6.41–20.57)	6	7.77 (2.85–16.91)	
35–49 years	650	20.86 (19.28–22.52)	31	18.88 (12.83–26.80)		42	20.81 (15.00–28.13)	31	18.88 (12.83–26.8)	
50–64 years	1,524	34.28 (32.58–36.05)	49	23.6 (17.46–31.20)		63	29.92 (22.99–38.28)	49	23.60 (17.46–31.2)	
65–79 years	1,607	53.08 (50.51–55.74)	22	28.59 (17.92–43.28)		49	50.38 (37.27–66.60)	22	28.59 (17.92–43.28)	
≥ 80 years	309	72.53 (64.66–81.08)	6	58.06 (21.31–126.38^)^		5	48.17 (15.64–112.42)	6	58.06 (21.31–126.38)	
Total	4,217	35.22 (34.16–36.30)	114	21.22 (17.51–25.50)^d^		172	27.38 (23.44–31.80)^d^	114	21.22 (17.51–25.50)^d^	
**Female**					1.53 (1.28–1.84)^b^					1.46 (1.16–1.83)^c^
**Age (years)**										
18–34 years	98	13.98 (11.35–17.04)	24	23.37 (14.97–34.77)		13	13.77 (7.33–23.55)	24	24.46 (15.67–36.4)	
35–49 years	437	13.27 (12.06–14.58)	24	10.38 (6.65–15.44)		32	12.74 (8.72–17.99)	24	10.38 (6.65–15.44)	
50–64 years	1,395	31.56 (29.93–33.27)	44	18.57 (13.5–24.93)		67	25.47 (19.74–32.35)	44	18.57 (13.5–24.93)	
65–79 years	1,810	49.95 (47.68–52.31)	27	27.87 (18.37–40.55)		56	45.53 (34.39–59.12)	27	27.87 (18.37–40.55)	
≥ 80 years	385	78.83 (71.16–87.12)	6	46.9 (17.21–102.09)		17	115.91 (67.52–185.58)	6	46.9 (17.21–102.09)	
Total	4,125	32.94 (31.94–33.96)^d^	125	18.37 (15.29–21.88)^d^		185	24.79 (21.35–28.63)^d^	125	18.37 (15.29–21.88)^d^	
CIR	0.20 (0.20–0.21^)^^d^		0.09 (0.07–0.11)^d^			0.16 (0.12–0.21)^d^		0.09 (0.08–0.11)^d^		

Abbreviations: CIR: cumulative incidence rate; aSHR: adjusted subdistribution hazard ratio; Ref., reference group; CI: confidence interval.

^a^ Based on Cox proportional hazard regression with competing risk analysis and adjusted for age, sex, selected comorbidities (diabetes mellitus, hypertension, coronary artery disease, acute myocardial infarction, anemia, hyperlipidemia, alcoholism, chronic obstructive lung disease and valvular heart disease) and the use of selected medications (angiotensin-converting-enzyme inhibitor, angiotensin II receptor blockers, Aldosterone antagonists, Beta-blocker, Diuretics, Digitalis glycosides and Nitrate + hydralazine).

^b^ *p* value < 0.001

^c^ *p* value < 0.01.

^d^ The comparisons of overall and sex-specific incidence rates and cumulative incidence rates between the HD and PD patients with and without matching were all statistically significant (*p*<0.001).

### Adjusted subdistribution hazard ratios of CHF related to the dialysis modality among the matched and unmatched dialysis cohort and the subgroup analysis based on various covariates

**Fig 3:**
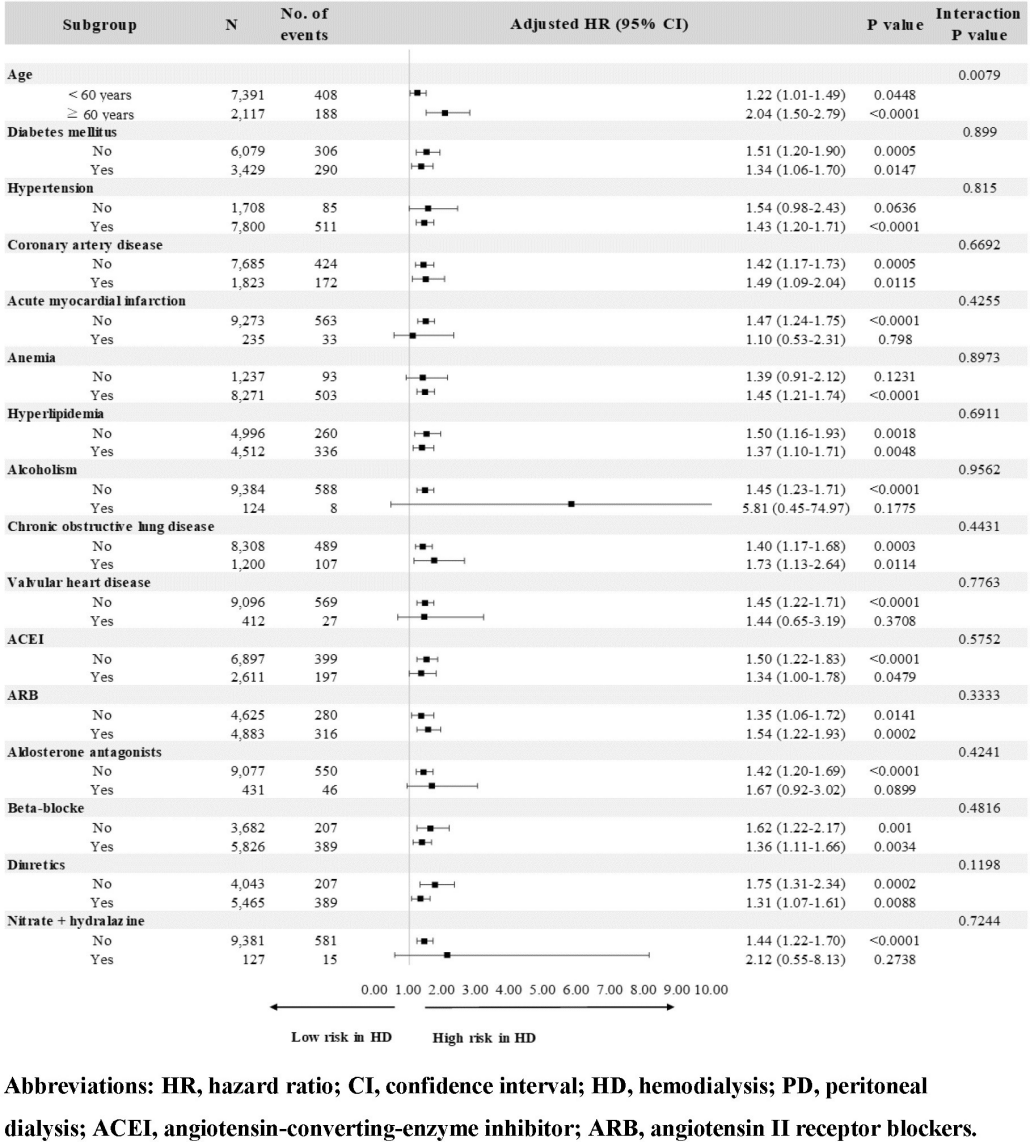
Stratified analysis of risk for CHF between matched pairs of hemodialysis and peritoneal dialysis patients using multivariable subdistribution hazard models.

The results concerning the risk of CHF between different dialysis modalities based on the multivariate subdistribution hazard models are also presented in Table 2. After adjustment for potential confounders, the HD patients were associated with a significantly higher risk of CHF when compared with their corresponding matched PD counterparts (adjusted HR: 1.45 [95% CI: 1.23–1.70], respectively). These results were similar in the analysis of the unmatched dialysis cohort (adjusted HR: 1.54 [95% CI: 1.35–1.75]). Stratified analyses according to each of the selected covariates were also performed in the matched cohort (Fig 3). When applying the appropriate interaction term in the multivariate models, only age was found to significantly modify the effect of dialysis modality on the risk of CHF (*P*_interaction_<0.0001). Age-specific analyses showed that HD was associated with significantly higher HR of CHF in patients aged ≥ 60 years (adjusted HR: 2.04 [95% CI: 1.50–2.79]) as compared to those aged under 60 years (adjusted HR: 1.22 [95% CI: 1.01–1.49]).

## Discussion

In this retrospective cohort study of dialysis patients, we compared the incidence of newly-diagnosed CHF requiring hospitalization after the initiation of maintenance dialysis therapy between matched pairs of patients receiving HD and PD. Our study results suggested that patients receiving HD had a higher risk of developing CHF than those receiving PD after adjustment for potential confounders and after taking the competing risk event (i.e., mortality) into consideration. The elevated risk of CHF associated with HD was even more evident in elderly patients aged ≥ 60 years as compared to younger patients, but it was quite homogeneous in patients characterized by various comorbidities and medication use. Therefore, our study results indicated that HD patients are at a higher risk of developing CHF and warrant more intensive surveillance programs and management for the prevention of CHF among dialysis patients. The K/DOQI guideline has suggested that all dialysis patients should receive echocardiogram study at a three-year interval, after the initiation of dialysis therapy [25]. So a shorter time interval, such as a one- to two-year interval, for heart failure surveillance program might be suggested for HD patients. More studies are still needed to clarify this issue. In addition, the New York Heart Association (NYHA), American College of Cardiology Foundation/American Heart Association (ACC/AHA) and K/DOQI guideline all suggested to follow up echocardiogram and serum biomarkers to see if there is any change in clinical status which is related to heart failure. This might also facilitate clinicians to confirm the diagnosis [19, 25]. Furthermore, patients with multiple CHF risk factors are likely to be candidates for PD therapy rather than HD therapy when initiation of dialysis therapy is considered.

Several studies have been conducted to evaluate the risk of CHF related to both HD and PD, but the findings from these previous studies have been inconsistent. Foley et al. reported that dialysis modality is not associated with increased risk of CHF [26], which was in contrast to the results of a study by Trespalacios and Wang et al. [16, 17]. The limited sample size and/or event numbers might explain the insignificant difference in risk of CHF between HD and PD [26]. The exclusive inclusion of the elderly population in the study of Trespalacios et al. might have resulted in an overestimation of the incidence rate of CHF in the dialysis population (71 vs 19.71–25.98 per 1,000 patient-years in our study) [17], which limited the generalization of their results. Since the occurrence of AMI will predispose a patient to the subsequent risk of CHF, the exclusion of patients with AMI will ultimately lead to underestimation of the incidence of CHF [16]. This observation was further confirmed by the lower incidence rates of CHF in the study by Wang et al. as compared with those found in our study (9.33–9.99 vs 19.71–25.98 per 1,000 patient-years, respectively) [16]. Furthermore, the selection of potential confounders in the study of Wang et al. was for *de novo* cardiovascular disease rather than CHF. This would further bias the estimation of propensity scores in logistic regression models and the HRs in Cox models if the primary outcome were to be changed to CHF. Therefore, we believe that the estimations from our study might be closer to the true risk of CHF induced by different dialysis modalities.

Several explanations might support the premise that HD is associated with higher CHF risk than PD. First, accumulated evidence suggests that HD can induce repeated myocardial ischemia and stunning, which might ultimately progress over time into fixed systolic dysfunction with distinct systemic hemodynamic consequences, including elevated cardiac troponin T levels, intra-dialytic hypotension, fatal arrhythmia, and even sudden cardiac death [27, 28]. One study throughout an 8-year surveillance program showed that PD patients can sustain better maintenance of various ultra-sonographic cardiovascular indices than HD patients [29]. Second, reperfusion injury, especially in the case of repetitive fluctuating hemodynamic status during the HD procedure, might be another potential mechanism leading to myocardial damage that could be attributable to HD [12]. Uremic toxins including indoxyl sulfate and *p*-cresol, which are risk factors for development of CHF, are lower in patients receiving PD than in those receiving HD [30, 31]. This phenomenon might be attributable to the better preservation of residual renal function, lower uremic toxin generation rate from intestinal microflora and metabolic disparity and the lower protein catabolic rate in peritoneal dialysis [32].

Practitioners need to be aware of various degrees of risk for developing CHF with known vascular comorbidity. Nevertheless, given the unfavorable impact of HD on the risk of CHF presented in this study, different time intervals for surveillance programs should be suggested to patients receiving different dialysis modalities. In addition, receipt of PD therapy for ESRD patients with higher risk of developing CHF should be considered, including those comorbid with diabetes (adjusted HR: 1.39, 95% CI [1.14–1.69]), coronary artery disease (CAD) (adjusted HR: 1.22, 95% CI [1.00–1.50]), hyperlipidemia (adjusted HR: 1.23, 95% CI [1.03–1.46]), and chronic obstructive pulmonary disease (adjusted HR: 1.29, 95% CI [1.04–1.60]). Furthermore, PD therapy is characterized with gentle removal of water and solutes, a lack of neurohumoral activation and stable hemodynamic status during treatment, as well as better removal of cytokine/chemokine, which would be beneficial for the cardiovascular system [33].

Dialysis patients are characterized with inadequate capacity of free water excretion and they are usually comorbid with fluid overload if they are not compliant with suggestions for fluid restriction or dialysis protocols. Although fluid overload is one of the risk factors for CHF, we would suggest that diagnosis of fluid overload is not equivalent to that of CHF, including mild CHF. The diagnostic criteria for heart failure suggested by either the NYHA/ACC/AHA should be mainly based on clinical symptoms related to heart failure [19]. The incorporation of non-invasive and invasive cardiac imaging (echocardiogram, radionuclide ventriculography, magnetic resonance imaging or cardiac catheterization) and serum biomarkers (natriuretic peptides) in the survey for patients with CHF could serve as useful markers for risk classifications or facilitating clinical judgements for diagnoses. The Acute Dialysis Quality Initiative XI Workgroup also developed a new functional classification for diagnosis of CHF specific to dialysis patients [34]. They incorporated another three core components in dialysis patients, including the criteria of response of congestive symptoms to ultrafiltration [34]. Moreover, they proposed a diagnostic pathway which might facilitate to differentiate the diagnosis of heart failure with preserved ejection fraction from pure volume overload by using the dynamic changes in echocardiographic parameters and the online hematocrit slope data during the process of volume removal [34]. If diagnoses of claim data are found to violate the current diagnosis guidelines [19, 25, 34], medical institutions might be fined up to 100-fold of the corresponding reimbursement healthcare expenditures according to the regulation of Taiwan NHI program. Therefore, the presence of fluid overload alone without any other concomitant CHF symptom or sign would not be easily classified into the diagnosis of CHF in our study.

Our study has several limitations. First, NHIRD does not collect all detailed information of potential risk factors for CHF, such as residual renal function, anthropometric parameters, nutritional status, dialysis adequacy, inflammatory markers, or cardiac function. Therefore, we were unable to control for these risk factors for CHF in the analyses. However, we first applied the propensity score matching method to minimize potential indication bias and to balance the potential confounders at baseline between the HD and PD patients. The Cox regression models were then constructed to adjust for these baseline confounders to reduce the residual confounding as much as possible. In addition, the quality control program for dialysis patients in the NHI system ensures that most dialysis patients maintain satisfactory levels of nutrition and dialysis adequacy. Since CAD, AMI, valvular heart disease, and cardioprotective medications can directly modify cardiac function, they could serve as surrogate markers of cardiac function and, at least partially adjust the effect of cardiac function in our Cox models. Nevertheless, we should highlight that the residual confounding caused by these unmeasured variables could still be present even though the two-step approach was employed in the current study. Second, the application of propensity score matching in the analysis may have helped improve the internal validity of our study results, but this might have been at the cost of compromising external validity. Less than 10% of the ESRD patients were selected in the final analysis after matching. To investigate whether our study results can be generalized to the entire dialysis population, we constructed another multivariate Cox model based on the unmatched HD and PD cohorts (Table 2), and the results essentially remained unchanged, which suggested the robustness and generalizability of our study conclusions. Third, the identification of comorbidities and primary outcomes were solely based on the ICD-9 codes; thus, potential misclassification bias might have existed in our study. However, disease misclassification of this type is likely to be non-differential, which would result in underestimation of the estimated relative hazard and should not be a valid argument against the observed association of dialysis modalities with CHF incidence noted in our study. Fourth, we excluded patients with dialysis mode-switching, which accounted for 21.9% (N = 1,218) of the PD patients and 1.95% of the HD (N = 1,344) patients, during study cohort enrollment. This exclusion was made in an attempt to assess the independent effect of different dialysis modalities on the risk of CHF. Because of the exclusion of patients with modality switching from our study, we could not clarify whether those with dialysis modality switching were associated with higher or lower incidence of CHF. Future studies are still needed to investigate this issue. Fifth, the identification of CHF was based on the NHIRD inpatient claims data. Therefore, our conclusion related to the risk of CHF might be more confined to advanced CHF, namely, the ACC/AHA stages C-D or the NYHA functional classes III-IV [19]. Since many large-scale clinical trials also have adopted “hospitalization for heart failure” as their outcome variables, such as TECOS [35] and SAVOR-TIMI 53 [36], we believe the outcome variable defined in our study, namely, hospitalization for heart failure, can serve as a representative cardiovascular outcome in clinical research. In addition, the accuracy of the diagnosis of CHF in hospitalization claims of the NHI database has been validated [37, 38]. The diagnosis of CHF admission exhibited a very high sensitivity (99%), leading to little likelihood of false-positive rate associated with the utilization of diagnostic codes of CHF in hospitalization claims [37]. Another study also proved the high sensitivity of the diagnosis of CHF either by review of cardiologists (96.3%) or by echocardiogram (91.3%) [38]. Sixth, roughly 25% of dialysis patients with prevalent CHF were excluded (Fig 1) in our study in order to establish the temporal association between dialysis modality and incidence of CHF in the design of cohort study. In addition, patients with mild CHF not requiring hospitalization would not be identified as reaching the endpoint in our study. We would underestimate the risk of CHF in dialysis patients if such an exclusion was not made at baseline or mild CHF was considered to be included in the outcome of our study. Seventh, our data derived from the NHIRD has a nine-year gap till now. Since medical care for CHF and dialysis patients has been improving over the past decade, it must have a beneficial effect on incidence of heart failure and life expectancy in dialysis patients. Consequently, the findings in our study might not be representative to current practice and the interpretation of our study results should be cautious.

In conclusion, our study demonstrated an increased risk of newly-diagnosed CHF requiring hospitalization in HD patients as compared to their propensity score-matched pairs of PD patients. Since a certain number of dialysis patients present risk factors for CHF, strategies for dialysis modality selection and intervals for active surveillance programs should be individualized for patients with different degrees of risk for CHF.

## Supporting information

S1 Table International Classification of Diseases, 9th Revision, Clinical Modification (ICD-9-CM) codes used to identify comorbidities. (DOCX)
