# Supplementary material for: A comparison of the risk of congestive heart failure-related hospitalizations in patients receiving hemodialysis and peritoneal dialysis - A retrospective propensity score-matched study
Source: PLoS One. 2019 Oct 1;14(10):e0223336. doi: 10.1371/journal.pone.0223336 (PMC6773217; doi:10.1371/journal.pone.0223336)
Supplement: S1 Table — (DOCX) [file pone.0223336.s001.docx]

**S1 Table. International Classification of Diseases, 9th Revision, Clinical Modification (ICD-9-CM) codes used to identify comorbidities**

| Comorbidities | ICD-9-CM |
| --- | --- |
| Acute myocardial infarction | 410, 412 |
| Diabetes | 250, 357.2, 362.0X, 366.41 |
| Hypertension | 401-402, 405 |
| Coronary artery disease | 414 |
| Valvular heart disease | 394-396, 424.0, 424.1, 424.2, 424.3 |
| Hyperlipidemia | 272.0-272.4 |
| Anemia | 280-285 or the prescription of erythropoietin stimulating agents |
| Chronic obstructive pulmonary disease | 491-494,496 |
| Alcoholism | 291, 303, 305.0, 357.5, 425.5, 571.0, 571.1, 571.2, 571.3, 980.0 |
